# Unraveling the Complex Relationship Triad between Lipids, Obesity, and Inflammation

**DOI:** 10.1155/2014/502749

**Published:** 2014-08-28

**Authors:** Shahida A. Khan, Ashraf Ali, Sarah A. Khan, Solafa A. Zahran, Ghazi Damanhouri, Esam Azhar, Ishtiaq Qadri

**Affiliations:** ^1^Department of Applied Nutrition, King Fahd Medical Research Center, King Abdulaziz University, P.O. Box 80216, Jeddah 21589, Saudi Arabia; ^2^Department of Medical Biotechnology, King Fahd Medical Research Center, King Abdulaziz University, P.O. Box 80216, Jeddah 21589, Saudi Arabia; ^3^National Brain Research Center, Manesar, Gurgaon District, Haryana 122 051, India; ^4^King Fahd Medical Research Center, King Abdulaziz University, P.O. Box 80216, Jeddah 21589, Saudi Arabia; ^5^Special Infectious Agents Unit, Biosafety Level 3, King Fahd Medical Research Center, King Abdulaziz University, P.O. Box 80216, Jeddah 21589, Saudi Arabia; ^6^Medical Laboratory Technology Department, Faculty of Applied Medical Sciences, King Abdulaziz University, P.O. Box 80216, Jeddah 21589, Saudi Arabia

## Abstract

Obesity today stands at the intersection between inflammation and metabolic disorders causing an aberration of immune activity, and resulting in increased risk for diabetes, atherosclerosis, fatty liver, and pulmonary inflammation to name a few. Increases in mortality and morbidity in obesity related inflammation have initiated studies to explore different lipid mediated molecular pathways of attempting resolution that uncover newer therapeutic opportunities of anti-inflammatory components. Majorly the thromboxanes, prostaglandins, leukotrienes, lipoxins, and so forth form the group of lipid mediators influencing inflammation. Of special mention are the omega-6 and omega-3 fatty acids that regulate inflammatory mediators of interest in hepatocytes and adipocytes via the cyclooxygenase and lipoxygenase pathways. They also exhibit profound effects on eicosanoid production. The inflammatory cyclooxygenase pathway arising from arachidonic acid is a critical step in the progression of inflammatory responses. New oxygenated products of omega-3 metabolism, namely, resolvins and protectins, behave as endogenous mediators exhibiting powerful anti-inflammatory and immune-regulatory actions via the peroxisome proliferator-activated receptors (PPARs) and G protein coupled receptors (GPCRs). In this review we attempt to discuss the complex pathways and links between obesity and inflammation particularly in relation to different lipid mediators.

## 1. Introduction

**Figure 1:**
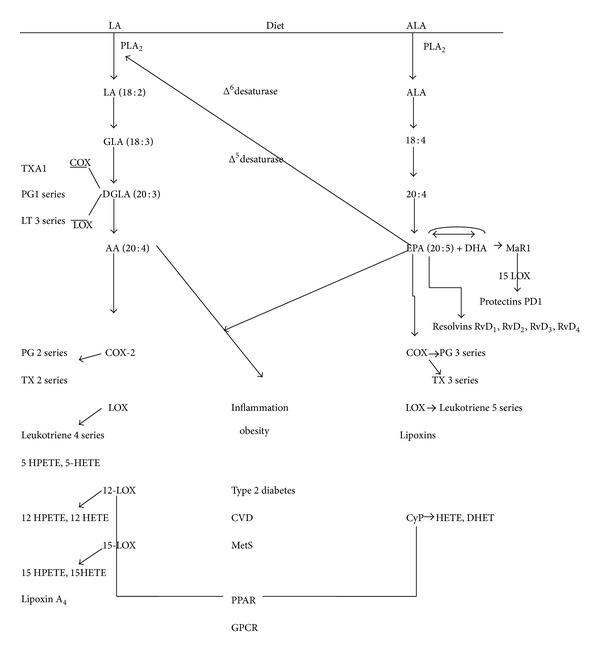
Major lipid influences in the inflammatory cascade. The different lipid molecules influenced by diet modulate the inflammatory cascade resulting in inflammation, obesity, cardiovascular disease, and metabolic syndrome. Dietary linoleic gets converted to the principally important proinflammatory arachidonic acid by the action of several enzymes. Further arachidonic acid by the action of COX, LOX gives rise to inflammatory responses. Linolenic acid on the other hand gets converted to eicosapentaenoic acid and docosahexaenoic acid which release anti-inflammatory resolvins, protectins, and maresins. Listed below are the abbreviations used in the figure: LA: linoleic acid; ALA: alpha linolenic acid; PLA2: phospholipase 2; GLA: gamma linolenic acid; DGLA: dihomo gamma linolenic acid; LT: Leukotriene; PGs: prostaglandins; TXA: thromboxane; COX: cyclooxygenase; LOX: lipoxygenase; RvD: resolvins; PD1: protectins; MaR: maresins; EETs: epoxyeicosatrienoic acids; HETEs: hydroxyeicosatetraenoic acids; HPETE: 5-hydroperoxy eicosatetraenoic acid; DHET: 14,15-dihydro eicosatrienoic acid; PPARs: peroxisome proliferator-activated receptors; GPCRs: G protein coupled receptors.

Obesity is a proinflammatory burning health issue recognized globally associated with increased risk of a cluster of risk factors which are representative of the metabolic syndrome. Energy uptake overrides expenditure causing a disruption of cellular functions augmenting insufficient appetite regulation, thereby leading to a chain of vicious events [1]. Obesity resulting in a disruption of insulin and lipid metabolic pathways gives rise to metainflammation, targeting critical organs and adversely affecting homeostasis. Obesity acts as a precursor to the metabolic syndrome which is a crucial stage to many an ailments exhibiting inflammation and has been contributory in disorders like arthritis, cancer, cardiovascular diseases (CVD), asthma, and Alzheimer's disease due to excessive and prolonged inflammatory responses [2, 3]. Far from being relegated to functions of energy storage, lipids have now emerged responsible for the production of different intercellular signaling molecules against inflammation. Eicosanoids, fatty acids, sphingolipids, and phosphoinositides manage crucial cellular processes, which include cell metabolism, proliferation, and apoptosis. Fatty acids influence inflammatory pathways by both extracellular receptor interactions and also by intracellular signaling mediators. The intracellular signals act through GPCRs in the endosomes and the nucleus [4]. The extracellular signaling from different nutrients, growth factors, and cytokines manages the lipid influencing enzymes like sphingomyelinase, phospholipases, phosphoinositide 3-kinase prostaglandin synthase, 5-lipoxygenase, and sphingosine kinase [5]. Imbalances in the complex network result in the pathogenesis of obesity and other health problems. Though significant contribution is displayed by the genetic makeup on the basal levels of these markers of inflammation, some influences are also exhibited due to inflammatory responses of fatty food intakes [6]. Among these of importance are the fatty acids, phospholipids, and lysophospholipids with the omega-6 and omega-3 fatty acids playing the roles of prominent proinflammatory fatty acid, anti-inflammatory fatty acids, and so forth [7]. Lipid mediated inflammation in obesity is strongly influenced by the cyclooxygenase (COX) and lipoxygenase (LOX) pathways and these inflammatory markers occupy an important position as they give insights into arising risk for CVD, diabetes, and other metabolic disorders (see Table 1). A targeted approach using lipids indicates that macrophages supplementation with PUFAs inhibits the metabolism of arachidonic acid (AA) thereby attributing potent anti-inflammatory effects. Short-term and long-term inflammatory stimulation resulting in COX pathways are shifted to the less inflammatory COX (PG3 and TX3), and the resolving LOX(LT5) pathways by the long chain polyunsaturated fatty acids (LC PUFA's) namely EPA & DHA, thereby offering protection against inflammation (See Figure 1). This results in the increased formation of the proresolving lipoxins and resolvins that perform as “stop-signals” of the inflammatory response promoting the resolution of inflammation. Recent researches show that their reduced presence in obese adipose tissue and their restoration by either exogenous means or feeding diets rich in omega-3-enriched products improve metabolic dysfunction as well as the inflammatory condition of adipose tissue [8]. The role of docosapentaenoic acid in the LOX pathway makes it worth reconsideration in the context of anti-inflammation [9]. Diets rich in fish oil PUFA's are also known to increase secretions of adiponectin and improve the skeletal muscle responses to insulin which is contrary to the high saturated fat diet resulting in insulin resistance [10]. LC PUFAs also regulate expression of genes through PPAR and nuclear factor kappa B (NF-*κ*B) transcription factors and via eicosanoid production by reducing the formation of proinflammatory cytokine from various cells including macrophages. Infiltrated macrophages are an integral part of the stromal vascular fraction of the adipose tissue and are implicated in the production of the proinflammatory monocyte chemoattractant protein 1, TNFα, and interleukin-6 (IL-6). This anti-inflammatory property of omega-3 fatty acids could be strategically employed in decreasing obesity-induced insulin resistance [11].

## 2. Proinflammatory Fatty Acids in Cell Signaling

Inflammation causing physiological changes is characterized by increases in certain mediators of lipids and peptide origin known as eicosanoids and the cytokines, respectively. Dietary intake of fats especially the 20 carbon arachidonic (AA) and eicosapentaenoic acid (EPA) polyunsaturated fatty acids influences the eicosanoid pathways as they are precursors to eicosanoid formation. Based upon cell signals, the production of different cytokines occurs in the leukocytes of humans in the presence of various eicosanoids. Oxygenation of polyunsaturated PUFAs occurs in the presence of three types of enzymes, namely, the cyclooxygenases, lipoxygenases, and cytochrome P giving rise to lipid mediators of inflammation also known as the oxylipins [30]. These oxylipins in turn activate the PPAR transcription factors that are ligand activated or signal via the GPCRs after diffusing through the plasma membrane [31]. The nature of the oxylipins is dependent on their double bond configuration and length. Their production depends on the type of PUFA oxidized and the enzyme acting on it thereby rendering it specific to certain receptors [32]. Most of the inflammatory molecules involved in cell signaling cascades are generated from the AA fatty acid cycle. Prostaglandins (PGs), leukotrienes (LTs), and thromboxanes (TXAs) collectively are termed as eicosanoids which are proinflammatory when derived from the omega-6 fatty acid and anti-inflammatory when derived from EPA anomega-3 fatty acid. Alterations in the plasma arachidonic: eicosapentaenoic acid ratio seems to be causative of dysfunctions in the metabolism of obese individuals [33]. The main player in the inflammatory pathway arising from dietary omega-6 fatty acid linoleic acid (LA) is AA. The pathway is described schematically in Figure 1. Initially LA gets desaturated by the action of delta 6 desaturase to form DGLA (20 : 3) via the formation of GLA which is acted upon by a delta 5 desaturase to form AA. DGLA as such is also a substrate for oxygenases [34, 35]. Dietary GLA gets elongated to DGLA by the action of elongases. This DGLA, which structurally closely resembles AA, slows the formation of inflammatory eicosanoids of the AA pathway and is found to be beneficial against inflammation [36]. Diet intakes as well as the action of delta 6 and delta 5 desaturases or elongases in the metabolic pathway are decisive in the development of inflammation and related disorders [37]. Therefore it is observed that major obesity as a result of improper diet is a predecessor to most of the complications of the metabolic syndrome. Action of delta 6 desaturases is dependent upon the 20 : 3/18 : 3 fatty acid ratio whereas delta 5 desaturases are dependent upon the 20 : 4/20 : 20 : 3 fatty acid ratio. This renders activity of fatty acid desaturases as possible biomarkers in the development of obesity, CVD, and metabolic disorders. It has been hypothesized that dysfunctions in the delta 5 and delta 6 desaturase levels could be a decisive marker in the progression of insulin regulation [38]. Owing to the fact that delta 5 desaturase activity is limited in human cells, the levels of DGLA or GLA through supplementation do not appreciably affect AA concentration in the monocytes, neutrophils, and the platelets [36]. DGLA is acted upon by COX-1 to form the series 1 PGs and TXA1 and by LOX to form the 3 series of LTs. Lysophospholipid acyltransferase and arachidonyl CoA act together to store AA obtained from the diet or by the desaturation of LA into the phospholipids of the cell membrane. The enzyme phospholipase 2 (PLA2) acts upon the cell membrane storage sites releasing free AA, the exact enzyme mediated mechanism of which yet remains to be elucidated. The ability of AA to diffuse into the nucleus and hinder with DNA transcription for cytokines or other hormones makes it prone to inflammation. The secretory PLA2 enzyme (sPLA2) has been characterized and found that two groups, namely, I and II, exist. Group I enzyme is present in the pancreas whereas group II is found in the synovial fluid and platelets thereby being implicated in inflammation. Expression of group II sPLA2 has been induced greatly by the cytokines, namely, IL-1, IL-6, and TNF alpha, in different cells which can be correlated to the production of PGs [39]. The enzyme cyclooxygenase (COX1 and COX2) acts upon DGLA or AA into series 1 and 2 autocrine lipid mediators also named prostanoids. PGD1, PGE1, PGF1, TXA1, PGD2, PGE2, PGF2, PGI2, and TXA2 represent these prostanoids (see Table 1). Depending upon the number of double bonds, residues of amino acids, and their ring structure, these moieties are rendered receptor specific. Characterization into D, E, and F is based upon their most potentially active ligand. Interaction of these prostanoids takes place with specific receptors that have been characterized, namely, EP, FP, IP, TP, and DP [40]. The cascade thus gets triggered. PGs derived by the action of COX on AA promote prothrombotic effects. TXA2 triggers the TP alpha receptor encouraging platelet aggregation. The prostaglandins PGI2, PGD2, and PGE2 bind to GPCRs on the platelet surface triggering a stop signal [41]. Recent studies find that PGE2 expresses a binding ability to both pro-EP3 and antithrombotic EP4 receptors which makes therapeutic studies more intriguing [42].

Obesity during pregnancy has been known to trigger imbalances in the glucose uptake pathway causing insulin resistance. Maternal obesity has been associated with elevated inflammation responses systemically as well as locally within the placenta and adipose tissue, mediating adverse outcomes [43]. The condition is exacerbated owing to the combination of insulin resistance as well as greater expression of placental inflammation by the cytokines, namely, IL-6 [44]. Changes are observed in the levels of protein hormones, acute phase proteins, and cytokines in the 2nd trimester of pregnancy with increases in leptin and high sensitive C-reactive protein (CRP) concentrations in terms of increasing basal metabolic index (BMI) and increased monocyte chemoattractant protein 1 in morbid cases of obesity [45]. Contractions mediated by the smooth muscle and endothelial cells are induced by TXA2. Dysfunctions in the TXA2 pathway are observed in obese pregnant women. TXA2 gives rise to TXB2 which is a stable moiety. Normally one would expect an altered expression of the TXA2 receptors (TXA2R) during contractions. But surprisingly BMI in pregnancy does not affect TXA2R in either the smooth cells or the endothelial cells. This is suggestive of alterations or deregulations of other mediators in the TXA2 pathway, yet to be properly understood.

Lipoxygenases on the other hand catalyze the hydroperoxidation of AA to form the 4 series LTs. These LTs are known to easily permeate through the plasma membrane and trigger the GPCRs. The action of 5-LOX on AA results in the production of LTA4 via the intermediate 5-hydroperoxy eicosatetraenoic acid (5-HPETE). This intermediate 5-HPETE gets immediately reduced to 5-hydroxy eicosatetraenoic acid (HETE) which is again acted upon by LOX to form LTA4, an epoxide that is unstable and is converted to LTB4. Four LOXs, namely, 3, 5, 12, and 15 LOX, have been identified depending on the carbon moiety oxidized. These LOXs represent and express 6AA genes. The role of 12 LOX and its derivative 12-HETE appears to be crucial in modulating adipogenesis and beta cell dysfunction as observed in insulin resistance. These leukotrienes produced promote bronchoconstriction in asthma and anaphylaxis which are subsets of illnesses with obesity as a precursor. LTA4 also conjugates with glutathione forming LTC4 which is cysteinyl leukotriene. The receptors Cys LT1 and Cys LT2 act on the surface of target cells causing bronchial contraction and migration of leukocytes. LTC4 are later converted by a number of enzymes to LTD4 [46]. Presence of LTB4 releases elevated concentrations of TNF α in human monocytes [47] and also influences the production of IL-1 and IL-6 [48]. TNF α has been implicated for its involvement in all stages of obesity leading to insulin resistance, hypertension, and the metabolic syndrome.

The role of TNF-α in inflammation, immunity, lipid metabolism, apoptosis, and insulin signaling is quite important. Increased TNF-α concentration is observed in circulating blood levels of obese individuals which decreases on losing weight. It is elemental in promoting the secretion of other proinflammatory cytokines (IL-6) and reducing adiponectin levels which is anti-inflammatory in nature. It promotes insulin resistance by the inhibition of the insulin receptor substrate 1 signaling pathway and also induces adipocyte apoptosis Although elevated levels of TNF-α, IL-6 and IL-1 are observed in the adipose tissue in conditions of obesity, only TNF-α concentrations are increased in the adipocytes [49]. In obese individuals, the macrophages in white adipose tissue are mainly responsible for the production of the proinflammatory cytokine TNF-α [50]. This propels activation of NF*κ*B causing oxidative stress and further enhancing the secretion of cytokine in peripheral tissues. The subcutaneous and adipose tissue seems to produce very minor quantities of TNF-α. The production of the anti-inflammatory cytokine adiponectin is decreased by TNF-α possibly due to its antagonist effect thereby resulting in deregulation of insulin signaling [51]. TNF-α is further responsible for dysfunctions in the endothelium leading to enhanced leukocyte adhesion,and activating the NFαB cascade, and inducing vascular cell adhesion molecule*-*1 (VCAM-1) expression in the endothelium [52]. Furthermore it elevates the atherogenic potential by promoting plaque formation and decreasing vasodilatation of blood vessels. It also stimulates the production of hepatic C-reactive protein (CRP) and interleukin-6 (IL-6) [53]. This pathway leads to CVD risks. The COX and LOX pathways are of prime clinical significance owing to being targets of the nonsteroidal drugs in case of inflammation, pain, and fever [54]. Of the two isozymes COX1 and COX2, only COX2 appears to be expressed at the inflammation site. However an imbalance in the ratio of COX1 derived TXAs: COX2 derived PGs promotes thrombosis [55], suggesting low dose aspirins to avoid risk of cardiovascular events [56]. Therefore the selective use of COX2 inhibitors as drugs has reduced over the years. Experimental evidences with nonsteroidal anti-inflammatory drugs aiming at COX2 selective inhibition have also resulted in low gastric tolerance [57].

## 3. Anti-Inflammatory Fatty Acids 

Gamma linolenic acid and the omega-3 fatty acids EPA and docosahexaenoic acid (DHA) belong to this anti-inflammatory category. GLA acts via DGLA to promote the anti-inflammatory eicosanoids. GLA and EPA act via displacement, competitive inhibition, or being counteractive. GLA increases the formation of DGLA resulting in lowered TXB2 and also a decreased conversion of AA to LTs. GLA also counteracts the PGE2 by promoting production of PGE1. Omega-3 PUFAs are essential components delivering health benefits in humans, and their deficiencies have been linked to chronic diseases [58]. DHA and EPA are omega-3PUFAs found in fish oils and are helpful in preventing or treating inflammatory diseases [59]. These PUFAs potentially act through multiple mechanisms which includes the proinflammatory eicosanoid and cytokine inhibition. EPA and DHA fatty acids inhibit a number of aspects of inflammation which include chemotaxis of leukocytes, VCAM-1 expression, leukocyte-endothelial adhesive interactions, production of eicosanoids, PGs and LTs from the omega-6 fatty acid AA, inflammatory cytokines production, and T cell reactivity [60]. Though animal studies suggest a protection against obesity by omega-3 fatty acids, only a few human studies have been well conducted [61]. In obese children elevated inflammation affects the vascular and endothelium wall which may be caused by a decrease in serum omega-3 concentrations. Conversely this condition can be reduced by supplementation of omega-3 fatty acids [62]. Dietary supplementation of omega-3 fatty acids has shown benefits in the control of inflammatory processes through mediators in humans [63]. EPA/DHA of marine origin expresses antiadipogenic effects during development of obesity and also decreases the accumulation of body fat. High level of dietary consumption of EPA/DHA has a beneficial effect regardless of the consumption of AA [64]. Experiments with omega-3 fatty acid treatment showed decreases in the lymphocytes and monocytes number and also decreases in the levels of TNF-α, IL-1*β*, and IL-6. Similar results are observed in healthy adults eating a Mediterranean diet [62]. Decreases in the level of the inflammatory factors TNF-α, IL-1*β*, and IL-6 in the adipose tissue improve endothelial function. Increased levels of omega-3 PUFA in serum phospholipids suggest an increased integration of omega-3 PUFAs into the membranes of circulating cells [62]. The omega-3 PUFAs seem responsible for the production of lipid signaling molecules of the anti-inflammatory type. These bioactive lipids arise from the parent omega-3 PUFA, namely, alpha linolenic acid, which is desaturated and elongated with an additional double bond to form EPA. This EPA by the action of elongase and delta 6 desaturase gets converted to a 24-carbon atom metabolite which undergoes beta oxidation to form DHA in the peroxisome [65]. COX converts EPA to the 3 series prostanoids, namely, PGD3, PGE3, and TXA3 [66]. These in turn permeate through the membrane binding to GPCRs in an autocrine or paracrine fashion. The potentiality of the ligand decides the reactivity with the specific receptors. The LOXs deoxygenate EPA to LTs, lipoxins, and hydroxyeicosatetraenoic acids (HETEs) 2 [67]. EPA competes with AA to form eicosanoids of the anti-inflammatory type and exhibits benefits in cases of inflammation and related vascular problems. Intriguingly, though DHA does not appear to be a COX substrate it gets oxygenated into the 14/11-OH and 17-OH metabolites by the action of 12- and 15-LOX [68, 69]. The heme protein cytochrome P450 (CyP450) exhibits specific absorbance at 450 nm on reduction, hence the name. Humans express 57CyPs which are subdivided into 18 families and 43 subfamilies depending upon the amino acid type and content. Role of the presence of CyP derived EET and HETE in platelets is not yet fully established [35]. CyP450 epoxygenases break down AA to epoxy eicosatrienoic acids (EETs) or HETE or omega-3 fatty acids of the epoxide type which improve the vascular endothelium, by inhibiting the action of NF*κ*B and downregulating COX and LOX cascades. The CyP 450 eicosanoids may serve as activators of PPARs in an autoregulatory manner thereby reducing the dysfunctions in adipocytes [7].

## 4. Other Lipid Mediators 

PGs, LTs, platelet-activating factor (PAF), lysophosphatidic acid, sphingosine 1-phosphate, and endocannabinoids are molecules classified as lipid mediators. They have a very important role in regulating the immune and defense system besides maintaining the homeostasis in living systems. The pathway leading to their synthesis is mediated by several enzymes, which get initiated by the deesterification of membrane phospholipids by phospholipase A2 or sphingomyelinase. Mostly they act by binding to associated receptors, which are members of the GPCR superfamily. Dysfunction in their pathway or deregulation of the enzymes leads to a variety of disease conditions. Besides these other inflammatory lipid mediators such as PAF, oxidized phospholipids, lysophosphatidic acid, and sphingosine1phosphate exist [70, 71]. Of late the role of endocannabinoids such as 2arachidonoylglycerol and anandamide in obesity is also gaining importance [72, 73]. PAF is an important lipid mediator, displaying significant role in immunity, inflammation, and obesity. PAF acetylhydrolase is a calcium independent phospholipase A2 that catalyzes the conversion of PAF to lyso-PAF. PAF-acetyl hydrolase (PAF-AH) also degrades oxidized phospholipids, which are formed during the oxidative modification of lipoproteins. Plasma PAF-AH deficiency is associated with atherosclerotic occlusive disease. PAF activity is found to be increased in obese hypercholesterogenic individuals when compared to lean individuals, which is indicative of the role of PAF in dyslipidemia and insulin resistance [74]. PAF may also act as a critical link in different diseases associated with insulin resistance syndrome. It has been found that, in adipocytes and preadipocytes, TNFα increases PAF synthesis and can also enhance adipocyte differentiation [75]. Sphingosine-1-phosphate might be a potential therapeutic target in obesity and metabolic dysfunction. Types 1 and 2 sphingosine kinases convert sphingosine into sphingosine 1 phosphate. Fingolimod (FtY720), a prodrug of a potent functional sphingosine 1 phosphate 1 agonist,, is currently undergoing clinical trials as an immunosuppressant in immune dysfunctions [76]. Sphingolipids also contribute to the prothrombotic and proinflammatory phenotype of obese adipose tissue. In diabetic* ob*/*ob* mice, plasma concentrations of total sphingomyelin, ceramide, sphingosine, sphingosine 1 phosphate, and adipose tissue sphingosine concentrations were found to be increased as compared to those in lean control mice. Sphingolipids therefore appear to play a very significant role in the pathogenesis of obesity-mediated cardiovascular and metabolic dysfunctions [71].

## 5. The Inflammatory Resolvers 

There are some other novel lipid mediators such as resolvins (Rvs), protectins, and maresins which are derived from these omega-3-PUFAs EPA and DHA. Oxygenated derivatives of DHA belonging to di- and trihydroxylated series and nonconjugated trienes are known to originate by the action of LOX. Accordingly few have been named resolvins as they speed up the inflammatory process of resolution [77]. Rvs and protectins are the emerging new families of mediators derived from EPA and DHA [58] possessing anti-inflammatory action [78]. Lipoxins (LXs) and Rvs are biosynthesized via the COX-2 or LOX pathways from omega-3 fatty acids. These LXs and Rvs in recent times have gained recognition because of their anti-inflammatory action, particularly in chronic disorders [63]. Rvs demonstrate anti-inflammatory and immunoregulatory action by reducing infiltration of the neutrophils and lowering the inflammatory response magnitude. Regulation of chemokine and cytokine axis, uptake and removal of polymorphonuclear (PMN), and generation of free radicals are also done by resolvins [79]. In addition, pharmacological studies suggest many other molecular targets like PPAR*γ* and GPCR120 for the anti-inflammatory action of omega-3 fatty acids [80]. Profiling of lipids has resulted in the identification of these Rvs and protectins, which have the ability to halt signals for infiltration of neutrophils and act as a marker in the inflamed tissue [81]. These protective mediators stop trafficking of leukocytes to the inflamed site thereby increasing vasodilatation and vascular permeability. They activate tissue debris clearance [82], regulate the production of cytokines, reduce the formation of reactive oxygen species, and also decrease the inflammatory response magnitude [83]. This process leads to the restoration of homeostasis in the inflamed tissue. The exact physiological function of the isomers of protectins like PDX is yet to be properly understood. Resolvins (RvD1) of the D series act by blocking PMN filtration and are much more resilient to metabolic inactivation (forming 17oxo RvD1) as compared to LX A4 [84]. Resolvins belonging to the D as well as E series have EPA as the precursor. RvE1 of the E series is a trihydroxylated form of the precursor fatty acid EPA which reduces the inflammation and blocks the migration of neutrophil in vivo [77]. It modulates the proinflammatory L-selectin and disrupts the TXA mediated platelet aggregation [85]. The other resolvin RvE2 also displays anti-inflammatory property by inhibiting zymosan-induced infiltration of PMN [79] Resolvins appear to inhibit IL-1*β* transcription through the action of TNFα and the migration of PMN leukocyte.

Protectins of the D series (PD) have DHA as their precursor. Through an enzymatic process via a 17S-hydroperoxide intermediate, 15 LOX forms the protectin, namely, PD1 (10, 17-diHDHA). The D series protectins are implicated in stroke and other neural diseases like Alzheimer's, asthma, and so forth, wherein they inhibit the formation of cytokines, proinflammatory gene expression, and the production of proinflammatory lipid mediators [86, 87]. Impairment in the formation of precursors to the resolution process and PD1 results in the inflammatory responses of the white adipose tissue in obesity [88]. Animal experiments support the action of PD1 in lowering accumulation of macrophage, decreasing blood glucose concentrations, enhancing adiponectin production, lowering the levels of proinflammatory cytokines [89], and getting expressed in a particular phenotype [90] as seen in obesity. Maresins are also DHA derived compounds that help in the resolution of the inflammatory status thereby maintaining homeostasis and promoting immunity and wound healing. Further researches are required to completely understand their individual complex roles in inflammation. Recently another novel compound 7, 14-dihydroxydocosa-4Z, 8, 10, 12, 16Z, 19Z-hexaenoic acid, which is a product of the 14-LOX pathway, has been characterized and seems bioactive in resolving the inflammatory status [79].

## 6. Modulation of Inflammatory Gene Expression by Peroxisome Proliferator Activated-Receptors 

Peroxisome proliferator-activated receptors (PPAR) play a very significant role in obesity, inflammation, and the metabolic syndrome apart from being apparent targets for complication related to the above life processes. They are principally a class of transcription factors which belong to the nuclear hormone receptor superfamily. Their functions involve regulation of gene expression by way of interactions with the retinoid X receptors. PPARs bind to the peroxisome proliferator response elements which are specific DNA sequences involved in gene regulation. Ligand-PPAR interaction leads to chromatin remodeling and recruitment of coactivators. This interaction results in initiation of DNA transcription [91, 92]. These PPARs control the regulation of inflammation and energy homeostasis. Due to their significant role they are the prime targets for drugs that control obesity, obesity-induced inflammation, insulin resistance, type 2 diabetes, CVD, and metabolic syndrome. Apart from being implicated in inflammation, PPARs seem elemental influencing the lipid and energy metabolism. Three isotypes of PPARs are so far known to exist. These are classified as PPAR-α, PPAR-*β* (also called PPAR-*δ*), and PPAR-*γ*. Their classification is based upon their expression on tissues and their development. Another factor considered is the distinct overlapping nature of lipid and eicosanoid ligands and their ability to activate each receptor [93]. Almost all PPARs are linked with lipid and fat metabolism besides bearing specific functions. PPARα was the first to be discovered among all PPARs. Its expression is more prevalent in tissues which have very high rates of the fatty acid *β* oxidation. Several organs of the body such as heart, kidney, muscle, liver, and cells of the arterial wall are central to its expression. Fatty acids, eicosanoids, 15-deoxy-delta prostaglandin J2, and fibrates appear to induce its function and expression. PPAR-α regulates expression of genes which are linked with apolipoprotein A-1 and also a major apolipoprotein of high density lipoprotein metabolism [94]. PPAR*α* also regulates inflammatory responses, particularly by reducing the effect of inflammatory genes involved in inflammation. Acute inflammation induced by cytokines and other molecules in the liver has been found to be reduced by PPAR activation. PPAR*α* illustrates its immunosuppressive effects in several ways [95]. It has the ability to interact with many proinflammatory factors which are involved in transcription, namely, signal transducer and activator of transcription 1, activator protein 1, and NF*κ*B [96]. PPAR*α* stimulates the expression of an inhibitory protein (I*κ*B*α*) which ultimately suppresses DNA-binding activity of NF*κ*B [97]. Furthermore PPAR*α* also reduces the activity of several proinflammatory transcription factors by confiscating the coactivator glucocorticoid receptor interacting protein-1 [98]. It inhibits the cytokine signaling pathways by downregulating the IL-6 receptor and upregulating soluble interleukin 1 (sIL-1) receptor antagonist [99], leading to reduced inflammatory responses. Several acute phase proteins which are found during elevated levels of inflammation get reduced by PPAR*α* activation using fenofibrate [100]. PPAR*α* controls inflammation of the adipose tissue through different pathways either by decreasing adipocyte hypertrophy linked with higher inflammatory tissues [101] or by directly regulating expression of genes potentially active in inflammatory pathways. Whether the anti-inflammatory effects of PPAR*α* in white adipose tissue are rendered effective by direct or indirect mechanisms is still not clear. PPAR*α* is also known to regulate genes and enzymes such as enoyl-CoA, acyl-CoA, thiolase, and medium chain acyl-CoA dehydrogenase that are involved in peroxisomal and mitochondrial beta-oxidation pathways. It also regulates genes involved in fatty acid transport and lipid metabolism. Activated PPAR-*α* leads to an increased cellular fatty acid uptake, which in turn induces a breakdown of triglycerides and fatty acids and lowers triglyceride and fatty acid synthesis by altering the transcription of several genes [102, 103].

PPAR-*γ* is the most widely studied among all PPARs and considered as a master regulator of adipogenesis. It is mostly found in adipose tissues, though traces are present in organs like skeletal muscles, colon, and the lungs [104]. PPAR*γ* production is induced by fatty acids and their derivatives which are linked with adiposity, insulin resistance and sensitivity, and functions of the placenta. It activates various transcription factors and coactivators including the steroid receptor coactivator-1. PGC1α and PGC1*β* are transcriptional coactivators that interact with PPAR*γ*. This allows an interaction with multiple proteins linked with the regulation of cellular metabolism, including cyclic AMP-response-element-binding protein and nuclear respiratory factors [105]. Three different isoforms of PPAR*γ* are known to exist of which PPAR*γ*1 is found in almost all tissues except the muscle. PPAR*γ*2 is more specific to adipose tissues and PPR*γ*3 is present mostly in the macrophages, surrounding the large intestine and adipose tissues. PPAR*γ* is a component of the thrifty genotype supposedly increasing a person's inclination towards insulin resistance [104]. Several eicosanoids and unsaturated fatty acids work as endogenous agonists of PPAR*γ*, while the antidiabetic drugs rosiglitazone and pioglitazone belonging to the group of thiazolidinediones act as synthetic agonists of PPAR*γ* [106]. The prostaglandin PGJ2 activates PPAR*γ* and conversely LTB_4_ gets activated by PPARα [107]. Genes linked with PPAR*γ* are found associated with lipid storage, adipocyte differentiation, and glucose metabolism. The genes affected with the above processes include aquaporin 7, CD36, phosphoenolpyruvate carboxykinase, lipoprotein lipase, and adiponectin [104]. Similar to PPAR*α*, PPAR*γ* is also involved in regulating the inflammatory response, especially in the area surrounded by macrophages. It regulates inflammation by its interaction with proinflammatory factors associated with transcription of NF-*κ*B, activator protein 1 (AP-1), and signal transducers and activators of transcription (STAT) [108]. Alternatively it acts by blocking the removal of corepressor complexes from inflammatory gene promoters, thereby resulting in inhibition of the inflammatory gene transcription [109]. It can be assumed that induction of PPAR*γ* might favor differentiation of adipocytes which results in a decreased inflammatory response of adipose tissue during obesity. Expression of PPAR-*γ* is found at several places across the body but its activity is prominent around components of the vascular system, placenta, and the large intestine. Altered mutations in PPAR-*γ* cause a variety of complications leading to insulin resistance, type 2 diabetes, hypertension, increased triglycerides, low high density lipoprotein level, and dyslipidemia [110] which are all clinical manifestations of the metabolic syndrome. PPAR*γ* activation inhibits monocyte and macrophage inflammatory activity by suppressing the activity of several nuclear transcription factors. This anti-inflammatory effect helps to reduce the risk of atherogenesis and cardiovascular disorders [111]. Compared to PPAR*α* and PPAR*γ*, the PPAR*β*/*δ* is much less studied. It is found in several tissues but weakly induced by lipids, PGs, and LTs. Once induced, it binds to DNA and regulates the transcription of genes. Its role is not clear due to its pervasive expression pattern, lack of specific ligands, and lack of availability of knock-out animal models. But recent research on PPAR *β*/*δ* −/− mice has given a strong stimulus to look for other functions executed by PPAR*β*/*δ* [112]. Mice lacking PPAR*β*/*δ* have shown several complications such as decreased wound healing, a reduction in adipose mass, and disturbed inflammatory reactions of the skin [112]. The exact role of PPAR*β*/*δ* during inflammation though is not yet clear. Some anti-inflammatory effect has been noted in macrophages which suggest a possible role in atherogenic inflammation. It appears to promote an anti-inflammatory gene expression profile that is associated with the supposedly anti-inflammatory B cell lymphoma-6 (BCL-6) protein [113]. This protein is a component of the PPAR*β*/*δ*-RXR*α* transcriptional complex in the unliganded state. Upon activation by ligand corepressors, PPAR*β*/*δ*-RXR*α* gets separated followed by PPAR*β*/*δ*-dependent gene transcription. The separated BCL-6 later acts as a repressor of the proinflammatory gene expression in macrophages [113]. PPAR-delta also acts as a downstream target of adenomatous polyposis coli, beta-cadherin associated protein, immunoglobulin transcription factor 2, and a tumor suppressor pathway associated with regulation of growth promoting genes such as c-Myc and cyclin-D1.

PPAR*β* displays its antiapoptotic function in keratinocytes by transcriptional control of the signaling pathways such as Akt/protein kinase B. PPAR-*δ* also acts as a downstream target of adenomatous polyposis coli which is the site of action of NSAIDs [114]. There are a variety of synthetic and natural products available which acts as agonists for the PPARs. These agonists are extensively used for the management of diabetes, insulin resistance, and lipid disorders which are offshoots of obesity. Of these 15d-PG is the most potent endogenous PPAR-*γ* agonist known. Insulin-sensitizing antidiabetic drugs like glitazones of the thiazolidinedione family are other PPAR-*γ* agonists which increase insulin-mediated glucose transport into the adipose tissue and skeletal muscle and are used as pharmacological ligands for the treatment of rheumatoid arthritis [115, 116]. NSAIDs are synthetic agonists of PPARα and PPAR*γ*. The fibrates, gemfibrozil, and fenofibrate are also used as PPARα agonists which limit cytokine-induced induction of the inflammatory functions of VCAM1 in response to TNF-α and tissue factor gene expression [117].

## 7. Supplementation Studies 

### 7.1. Human Studies

Inflammatory molecules are released by the adipose tissue in the obese state. A potential pathway involving omega-3 fatty acids may include the uptake of DHA in inflamed cells in place of AA, so that lesser amounts of substrate are made available for the generation of eicosanoids [118]. Dietary alterations with LCPUFAs could influence the levels of circulating inflammatory markers in hypercholesteremic subjects. Effects of AA on the formation of prostaglandins were measured in an experimental study on healthy young men aged 20–38 years. Supplementation of 1.5 g AA per day for a period of 7 weeks exhibited elevated levels of PGE2 and the leukotriene LTB4 with no significant changes in the TNFα as well as IL1 and 6 [119]. Studies by Caughey et al. [120] showed that supplementation of 13.7 g of ALA per day for a period of four weeks to healthy volunteers decreased TNFα by 27% and IL-1 by 30% (see Table 2). Fish oil containing 9 g of EPA and DHA together per day caused a decreased generation of TNFα by 70% and IL1 BY 78%, respectively. These results suggest that EPA and DHA are more effective than ALA in exhibiting the anti-inflammatory effects [120]. In one study, the diet of two groups of human subjects was kept low in cholesterol and saturated fats and varied in their PUFA content. The ALA diet is comprised of 6.5% energy from ALA and the LA diet is comprised of 12.6% energy from LA, respectively. The ALA group showed decreases in CRP with *P* value < 0.01 as compared to the LA group with *P* value of 0.08. Also VCAM-1 was decreased by 16% (*P* value < 0.01) as compared to 3.1% in the LA group. An inverse association between changes in EPA levels and levels of CRP and VCAM-1 was observed on consumption of the ALA diet. ALA thereby favorably reduces inflammation and lowers CVD risk [18] Dietary intervention with 15 mL of ALA rich flaxseed oil for a period of 3 months in a group of 50 subjects exhibiting dyslipidemia, decreased CRP levels by 38% (*P* = 0.0008), and IL-6 levels by 10.5% (*P* = 0.01) independent of the changes in lipids [17].

Though restriction of energy resulting in weight loss is the primary intervention implemented to slow down and reverse the metabolic complications due to obesity, reducing inflammation through omega-3 PUFAs of marine origin of the adipose tissue is now being explored. Around 70% of the adipose tissue is composed of lipids. The omega-3 PUFAs are stored in the adipose tissue which account for 15–25% of the body weight in lean subjects and up to more than 50% in obese individuals [121]. Sixty-three hypertensive patients with a high BMI were supplemented with 3.65 g of marine origin omega-3 fatty acids (EPA and DHA) which did not only show a reduction of obesity (26% weight loss), but also exhibited a decrease in plasma triglyceride levels by 38% (*P* value < 0.001) and increases in HDL cholesterol by 24% (*P* value = 0.04) [23] (see Table 2). In a randomized 3-year study involving 563 elderly hyperlipidemic men, 2.4 gm/day of omega-3 marine PUFA appears to reduce soluble intercellular adhesion molecule 1 levels with a *P* value of < 0.001. This is indicative of lowered endothelial cell activation, assisting in the decreased rate of atherosclerosis [122].

### 7.2. Animal Studies

In a study by Ruzickova et al., a high fat diet that induced obesity in C57BL/6J mice was reduced when the EPA DHA content was increased from 1 to 12% (wt/wt) thereby restricting hypertrophy as well as hyperplasia of adipocytes rats [123]. Animal experiments with C57BL/6J mice showed that, in comparison to a low fat diet comprising 10% energy from fat, the group of mice fed a high fat EPA diet comprising 45% energy from fat which prevented them from becoming obese [124].

Beneficial effects of EPA have been further corroborated. Overweight Wistar male rats were divided into two subgroups, one of them receiving a standard high fat diet and the other an EPA inclusive diet for a period of five weeks. EPA was given at a concentration of 1 g/kg body weight. Increases in body weight as well as fat mass which were induced by the high fat diet reduced consumption of EPA (*P* = 0.09) with an increase in production of leptin (*P* < 0.05). Also the TNFα levels were found to be elevated only in the high fat diet group (*P* < 0.05) as compared to the EPA group (*P* < 0.01) [125].

## 8. Conclusion

It has been observed that overnutrition causes not only a storage of calories in the form of triglycerides, but also a hypertrophy of the adipocytes which subsequently produce increasing amounts of prostaglandins, raises macrophage infiltration, and results in elevated levels of the proinflammatory cytokines [126]. Association of obesity with a chronic low-grade inflammation of the adipose tissue results in co-morbidities like type 2 diabetes, CVD, gallbladder disease, stroke, psychosocial problems, osteoarthritis and certain cancers [127]. Obesity also acts as a trigger to hypertriglyceridemia and hyperglycemia which pose a risk to the development of chronic liver disease [128, 129] and appears to be a forerunner towards the metabolic syndrome. The chronic inflammatory response caused by obesity and enhanced production of IL-6 and TNFα may also increase the risk of many cancers [130]. Breast cancers exhibit increased eicosanoid concentrations of PGE2 and products of LOX action and seem to promote the growth of these cancers aggressively [131]. Understanding these diet related inflammation, the complex cascades and gene interactions in depth may contribute to growth in the challenging field of lipid science due to the growing burden of obesity worldwide in the last few decades. These inflammatory markers and their pathways occupy an important position as they give insights into assessing risk for CVD, diabetes, and metabolic disorders. A persistent inflammatory presence in obesity subsequently results in a “resolution deficit” by the anti-inflammatory omega-3 fatty acids thereby preventing the return to tissue homeostasis. Dietary fatty acids exhibit potential ability in regulating the inflammatory gene expression. Novel insights show the role of resolvins and protectins as displaying potent properties of anti-inflammation and possibly working as endogenous “stop signals” [132]. These omega-3-PUFAs, especially the ones with long chain lengths, have been shown to exert anti-inflammatory effects [133, 134] through modulation of transcription factors like NF*κ*B and PPAR*γ* activation [95]. Associations between dietary fat intakes and the outcomes of pathway dysfunctions can be drawn based upon the inflammatory signals produced [135] suggesting that diet based approaches could possibly act as target molecules in the prevention and treatment of inflammation in obesity and related metabolic disorders. Manipulating the scales of pro- and anti-inflammatory moieties could also help in understanding the systems and develop therapies. Targeting these inflammatory markers through lipid manipulations could slow down the progression of obesity to the metabolic syndrome. Complex interactions amongst the different neurotransmitters in the brain eventually determine and regulate food intake. Researches show an association between 12-month breast-feeding and reduced incidences of obesity. Breast milk being a rich source of long chain polyunsaturated fatty acids (LCPUFAs) inhibits proinflammatory cytokine production and enhances insulin receptors in different tissues. A rich presence of PUFAs in large quantities is observed in the brain. Their participation in neurogenesis might play a crucial role in the development of brain and its function suggesting one of the reasons for obesity to be a result of insufficient breast feeding, which culminates in marginal deficiency of LCPUFAs during the critical stages of brain development. This seems to cause an imbalance in the neurotransmitters and their receptors, which ultimately leads to a decrease in dopamine and insulin brain receptors [136]. Animal experiments show decreased circulating levels of inflammatory markers TNFα, IL-6, and C-reactive protein in PUFA fed rats as compared to saturated fatty acid fed rats. Also a noticeable downregulation of TLR 4 protein, which is a significant modulator for proinflammatory cytokine levels, has been observed [137]. Inflammatory markers affecting PPARs and GPCRs thus may have greater potential as means to increase risk assessment in persons requiring cardioprotective drug therapies, as well as for those in need of therapeutic lifestyle changes. Thus carefully designed observational studies examining the link between lipid metabolism and inflammation could open up new facets for developing treatments of targeted disease prevention in high risk individuals.

Guarding against metabolic dysfunctions due to obesity may increase effective strategies and interventions providing better management of the seemingly innocuous phenotype of obesity. The unprecedented increases in omega-6 refined oils with simultaneous decreases in omega-3 oil intakes in the past few decades have created havoc to the human bodily functions. Awareness on the lethal link between the obesity and the metabolic syndrome would caution an individual to take positive steps towards weight reduction which has profound benefits in timely improvements of the inflammatory responses. Encouraging consumption of LC -omega-3 PUFAs would assist return of the adipose tissue towards homeostasis. Though certain pharmacological agents do address these altered dynamics, a thoughtful perception of the proinflammatory lipids holds great promise of targeted treatments in the future [138]. Table 2 gives us an insight into certain supplementation studies with different dietary interventions [12–16, 19–22, 24–29]. Further studies are necessary to elucidate and unravel the missing links in the pathways to clarify our understanding of inflammation in obesity leading to vital dysfunctions.

## Figures and Tables

**Table 1:** Lipid signaling molecules and their actions in obesity related inflammation.

Inflammation				Resolution			
Omega-6 fatty acid mediated marker				Omega-3 fatty acid mediated marker			
Fatty acid	Enzyme	Proinflammatory marker	Action triggered	Fatty acid	Enzyme	Anti-inflammatory marker	Action triggered
		PGD1	Proarrhythmic			PGD3	Antiarrhythmic
		PGE1				PGE3	
	COX				COX		
		PGF1				PGF3	Antithrombotic
		TXA1				PGI3	Antiplatelet
						TXA3	aggregation
DGLA				EPA		TXB3	
							
		LTA3				LTA5	Dilation of blood vessels
	LOX	LTC3	Constriction of blood			LTB5	
		LTD3	vessels		LOX	LTC5	
						LTD5	
						LTE5	

	COX	PGD2 PGE2 PGF2 PGI2 TXA2	Prothrombotic, platelet aggregation via thromboxane A2 receptor			LIPOXINS	Act upon PPARs, GPCRs
						HETE	Inhibits free radical production
				
				
					
							
							
AA				EPA	CyP		
							
		5HPETE—5HETE—EETs				DHETs	Improve vascular tone and renal function and reduce hypertension
			Leukocyte activation				
	LOX	LTA4				Resolvins	Blocking PMN filtration, by inhibiting TNFα
		LTB4	Vasoconstriction, bronchoconstriction via the BLT1 and BLT2 receptors			Protectins	
		LTC4				Maresins	
		LTD4					
		LTE4					

Arachidonic acid (AA), eicosapentaenoic acid (EPA), docosahexaenoic acid (DHA), gamma linolenic acid (GLA), DGLA-dihomo gamma linolenic acid (DGLA), tumor necrosis factor alpha (TNF*α*), leukotriene (LT), prostaglandins (PGs), thromboxane (TXA), lipoxins (LXs), resolvins (Rvs), D series protectins (PDs), cyclooxygenase (COX), lipoxygenase (LOX), and hydroxy eicosatetraenoic acids (HETEs).

**Table 2:** Summary of supplementation studies examining the effects of dietary interventions with different fatty acids on biomarkers of inflammation.

Different dietary combinations	Duration of study	Changes in inflammatory biomarkers	Sample size	Reference
Control diet (30% fat) or experimental diets (39% fat with 8% substitution of oleic acid, trans fatty acid, saturated fatty acid, stearic acid, or trans + stearic acid)	5 weeks	Increase in CRP and E-selectin levels with *trans*fat diet as compared with control; increase in fibrinogen in stearic acid diet versus control; no difference in any marker between oleic acid diet and control (*P* < 0.05)	50 health adult males	[12]

Experimental diets (30% fat) two-thirds fats substituted with soybean oil, semiliquid margarine, soft margarine, shortening, stick margarine, or butter	35 days	No effect on CRP with any dietary fat type (*P* > 0.05)	36 moderately hypercholesterolemic adults	[13]

High-fat diet (59% fat) or high carbohydrate diet (73% carbohydrates), with or without antioxidants	1 week apart, 4-day study	Increase in IL-6, TNF-*α*, ICAM-1, and VCAM-1 in healthy and diabetic subjects with high-fat meal; increased levels only in diabetics with high-carbohydrate meal (*P* < 0.05)	20 type 2 diabetic patients and 20 matched healthy subjects	[14]

Experimental diets (30% fat) two-thirds fats substituted with soybean oil, soybean oil based stick margarine, or butter	32 days	Increase in IL-6 and TNF*α*, with stick margarine diet versus soybean oil diet (*P* < 0.05)	19 moderately hypercholesterolemic adults	[15]

Low cholesterol/low-saturated fat diet 30% fat, 5% saturated fat, cholesterol <200 mg	8 weeks	Decrease in CRP levels in hypercholesterolemic patients as compared to baseline (*P* < 0.05)	35 patients with primary hypercholesterolemia and 15 normal control subjects	[16]

15 mL linseed oil (8 grams ALA) or 15 mL safflower oil (11 grams LA)	12 weeks	Decrease in CRP, SAA, and IL-6 in ALA group; no effects with LA (*P* < 0.05)	76 male dyslipidemic patients	[17]

ALA diet (6.5% ALA, 10.5% LA), LA diet (12.6% LA, 3.6% ALA), or AAD–low carb diet (7.7% LA, 0.8% ALA)	6 weeks	Decrease in CRP, VCAM-1, and E-selectin in ALA group versus LA; decreased ICAM-1 in ALA and LA groups versus AAD (*P* < 0.05)	23 hypercholesterolemic adults	[18]

ALA-enriched (15% ALA, 46% LA) or LA-enriched (58% LA, 0.3% ALA) margarine	2 years	Decrease in CRP in the ALA group versus LA (*P* < 0.05) group	103 moderately hypercholesterolemic adults	[19]

4 grams EPA + DHA, with or without atorvastatin (40 mg)	6 weeks	Decreased CRP and IL-6 with fish oil + atorvastatin, but not with fish oil alone (*P* > 0.05)	48 obese individuals and 10 lean normolipidemic men	[20]

1.5 grams EPA + DHA, with or without 800 IU *all-rac α*-tocopherol	12 weeks	No effects on biomarkers of inflammation (*P* > 0.05)	80 healthy subjects	[21]

1.35 grams of EPA + DHA or placebo capsules	6 weeks	No effects on biomarkers of inflammation (*P* > 0.05)	11 obese men	[22]

4 grams, DHA, or placebo	6 weeks	No effects on biomarkers of inflammation (*P* > 0.05)	51 treated-hypertensive type 2 diabetic subjects	[23, 24]

1.5 grams EPA + DHA or placebo	12 weeks	No effects on biomarkers of inflammation (*P* > 0.05)	43 men and 41 postmenopausal women	[25]

1.33 grams EPA + DHA or 2.56 grams EPA + DHA, or placebo	5 weeks	Decreased CRP and IL-6 with fish oil versus placebo (*P* < 0.05)	30 postmenopausal women using HRT	[26]

3.4 g CLA or 3.4 g purified *t*10*c*12 CLA, or placebo D	12 weeks	Decreased CRP with *t*10*c*12 CLA supplementation versus placebo (*P* < 0.01)	60 men with metabolic syndrome	[27]

3.0 grams CLA isomer mixture or placebo	8 weeks	CLA decreased fibrinogen (*P* < 0.01); no effects on CRP, IL-6 (*P* > 0.05)	32 adults with diet-controlled type 2 diabetes	[28]

4.2 grams CLA isomer mixture or placebo	12 weeks	Increase in CRP with CLA mixture versus placebo (*P* < 0.01), no effects on TNF-*α* and VCAM-1 (*P* > 0.05)	53 healthy volunteers	[29]

Polyunsaturated fatty acid (PUFA), arachidonic acid (AA), eicosapentaenoic acid (EPA), docosahexaenoic acid (DHA), gamma linolenic acid (GLA), DGLA-dihomo gamma linolenic acid (DGLA), cardiovascular disease (CVD), tumor necrosis factor alpha (TNF*α*), IL-6-interleukin-6 (IL-6), cyclooxygenase (COX), long chain polyunsaturated fatty acids (LC PUFA's), linoleic acid (LA), C-reactive protein (CRP), vascular cell adhesion molecule*-*1 (VCAM-1), toll-like receptor 4 (TLR 4), and activator protein 1 (AP-1).

## Abbreviations

PUFA: Polyunsaturated fatty acid
AA: Arachidonic acid
EPA: Eicosapentaenoic acid
DHA: Docosahexaenoic acid
GLA: Gamma linolenic acid
DGLA: DGLA-dihomo gamma linolenic acid
CVD: Cardiovascular disease
TNFα: Tumor necrosis factor alpha
(PLA2): Phospholipase 2
LT: Leukotriene
PGs: Prostaglandins
TXA: Thromboxane
LXs: Lipoxins
IL-6: IL-6-interleukin-6
Rvs: Resolvins
PDs: D series protectins
COX: Cyclooxygenase
LOX: Lipoxygenase
HETEs: Hydroxy eicosatetraenoic acids
PPARs: Peroxisome proliferator-activated receptors
GPCRs: G protein coupled receptors
PAF: Platelet-activating factor
CyP450: Cytochrome P450
PMN: Polymorphonuclear
NSAIDs: Nonsteroidal anti-inflammatory drugs
LC PUFA's: Long chain polyunsaturated fatty acids
LA: linoleic acid
CRP: C-reactive protein
BMI: Basal metabolic index
VCAM-1: Vascular cell adhesion molecule-1.
